# Impact of COVID-19 pandemic on surgical cancer care disparities according to socioeconomic status

**DOI:** 10.1016/j.puhip.2026.100818

**Published:** 2026-06-03

**Authors:** R.M.G. van Vuren, R. van den Hoek, S. Kruijff, D.J. Heineman, N.F.M. Kok, R.J. Swijnenburg, M.R. Visser, S.S. Gisbertz, T.E. Hendriks, J. de Vos-Geelen, W.Y. van der Plas, M.W.J.M. Wouters

**Affiliations:** a The Netherlands Cancer Institute, Department of Surgical Oncology, the Netherlands; b Leiden University Medical Center, Department of Biomedical Data Sciences, the Netherlands; c Dutch Institute for Clinical Auditing, the Netherlands; d Academic Medical Centre Groningen, Department of Surgery, the Netherlands; e Academic Medical Centre Groningen, Department of Nuclear Medicine and Molecular Imaging, the Netherlands; f Karolinska Institutet Stockholm, Department of Molecular Medicine and Surgery, Sweden; g Amsterdam UMC, Department of Cardio-thoracic Surgery, the Netherlands; h Cancer Centre Amsterdam, Amsterdam UMC, VU University Medical Center, the Netherlands; i Amsterdam UMC location University of Amsterdam, Department of Surgery, the Netherlands; j University Medical Center Utrecht, Department of Surgery, the Netherlands; k Maastricht University Medical Centre, Div. Medical Oncology, Dept. Internal Medicine, GROW – Research Institute for Oncology & Reproduction, the Netherlands

**Keywords:** Socioeconomic status, COVID-19 pandemic, Healthcare scarcity, Population-based, Surgical oncology

## Abstract

**Objectives:**

This study aimed to examine whether healthcare scarcity during the COVID-19 pandemic amplified prior socioeconomic disparities in access to Dutch surgical cancer care.

**Study design:**

Retrospective population-based cohort study.

**Methods:**

Patients who underwent surgery for lung, oesophageal, gastric, pancreatic, or malignant liver tumours were identified in national quality of care registries. A pandemic cohort (15-03-2020 to 24-04-2022) was compared with a pre-pandemic cohort (2018–2019). Socioeconomic status (SES) was based on postal-code–level scores. Primary outcome was the difference in SES distribution between cohorts. Secondary outcomes were time to treatment and likelihood of stage III disease. Multivariate logistic regression incorporated all variables associated with tumour stage.

**Results:**

A total of 29,792 procedures were analysed (14,208 pandemic; 15,584 pre-pandemic). SES distribution was similar between periods (median 0.057 vs 0.059, p = 0.7). Median time to treatment was 27 days in both cohorts (p = 0.10) with no SES-related differences. Rates of surgery for stage III were comparable across SES groups. Adjusted analyses showed no association between SES and tumour stage in either cohort (pandemic aOR 0.88, 95% CI 0.74–1.03).

**Conclusions:**

Despite pressure on healthcare during the pandemic, access to surgical cancer care remained equitable across SES groups in the Netherlands.

**What this study adds**

- Unlike most international studies reporting widened socioeconomic disparities during the COVID-19 pandemic, this population-based cohort study in the Netherlands found no evidence of increased inequalities in surgical cancer care, most likely explained by the prioritization of cancer care during the pandemic.
- The findings suggest that near-universal health coverage and the absence of parallel public-private systems (as seen in the Dutch model) may mitigate the impact of healthcare scarcity on inequalities.

**Implications for policy and practice**

- For policymakers: Health systems aiming to maintain equity during future crises should strive for robust universal coverage and minimize regional or socioeconomic fragmentation in service availability.
- For healthcare workers: In crises or situations of healthcare scarcity, increased attentiveness to the needs of marginalized populations is vital to safeguard equitable access to care.
- For researchers: To enhance our collective understanding of the factors that make healthcare systems resilient to threats against equity of access, publication of findings that demonstrate no disparities is as important as those that report significant disparities.

## 1 Introduction

An increasing focus is being directed towards the disproportionate effects of cancer on people of lower socioeconomic status across the entire spectrum of cancer care, from screening and diagnostics to overall survival. Socioeconomic status (SES) describes the position of individuals on a hierarchical social structure based on wealth and access to resources. In relation to healthcare, Shavers [1] defines SES as “an attempt to capture an individual's or group's access to the basic resources required to achieve and maintain good health”.

Socioeconomic differences in cancer incidence, treatment, and outcomes have been observed worldwide. For decades, lower socioeconomic groups have had a higher incidence of cancer in the USA [2]; and they frequently present with more advanced stages of disease at time of diagnosis [3]. A large European study with multiple cancer types reported that groups of lower-educated individuals had higher mortality rates, particularly for tobacco/infection-related cancers [4]. It is evident that SES disparities are a major concern in cancer care.

Ranking third on the global Healthcare Access and Quality Index [5], the Dutch healthcare system is considered to perform well in terms of equal access to services, yet disparities persist. The Dutch National Institute for Public Health and the Environment concluded that differences in access between socioeconomic groups are mainly linked to cultural rather than financial aspects [6]. A recent report by the Netherlands Comprehensive Cancer Organisation [7,8] showed that higher-income patients present with more favourable disease stages at diagnosis, partly due to greater participation in screening programs, which is in turn associated with higher health literacy. Lower-SES patients were less likely to receive cancer treatment and reported poorer quality of life after diagnosis.

It is unclear whether healthcare scarcity during the COVID-19 pandemic worsened existing disparities in Dutch cancer care. A surge in demand for care and subsequent redeployment of operating room personnel and facilities to intensive care, illness among healthcare workers, physical distancing requirements, and a shortage of material resources all contributed to an unprecedented situation of healthcare scarcity at the national level. Chronic healthcare scarcity is expected to become more frequent due to an ageing population, staff shortages, and potential future pandemics. These conditions may heighten barriers to accessing care— for example, patient hesitancy to seek medical attention, and reduced capacity in outpatient clinics and hospital wards. Examining SES-related inequities under scarcity is essential for informing policies and strategies to mitigate future disparities.

This study investigates three hypotheses regarding the impact of healthcare scarcity during the COVID-19 pandemic on surgical cancer care access across socioeconomic status (SES) groups. First, if patients with lower SES face greater barriers, they will be underrepresented in SES distribution. Second, these barriers may delay presentation, resulting in more advanced disease at diagnosis compared to pre-pandemic. Third, under system pressure, some patients may seek to minimize their waiting times; however, lower SES patients may lack the health literacy to do so, leading to longer time to treatment. We aim to assess SES distribution, tumour stage at diagnosis, and time to treatment initiation in Dutch patients with lung, hepato-biliary and upper gastrointestinal cancers.

## 2 Methods

### 2.1 Study design and population

This population-based cohort study included all patients registered at the Dutch Institute for Clinical Auditing (DICA) who underwent surgery for lung cancer, oesophageal and gastric cancer, pancreatic and periampullary cancer, and malignant liver tumours. The quality registries at DICA are nationwide, mandatory, prospectively maintained, and accredited by scientific boards; data quality is assured and all Dutch hospitals participate in them [[9], [10], [11], [12], [13]]. Data from the pandemic cohort (15 March 2020 to 24 April 2022) were compared with those from a pre-pandemic cohort (1 January 2018 to 14 March 2020). As data was handled anonymously, no ethical approval was required according to Dutch law.

### 2.2 Definitions

Socioeconomic status (SES) was determined on the neighbourhood level using the patients’ four-digit postal code. These postal code areas have been assigned an average SES score based on household level of education, labour participation and financial welfare in 2019 by Statistics Netherlands (a government agency known as CBS) [14]. The SES scores range between −1 and 1, with 0 as the median in the general Dutch population. To divide patients into SES categories, three groups were defined based on SES score deciles in the general Dutch population: low (lower 3 deciles), medium (middle 4 deciles) and high (upper 3 deciles).

Time to treatment initiation was calculated as the number of days between histopathological diagnosis (in case of upper gastro-intestinal cancer) or decision on the treatment plan (multidisciplinary tumour board meeting), and either start date of neo-adjuvant treatment or date of surgery. Patients undergoing urgent surgery within 72 h after diagnosis were excluded from the time to treatment analysis. In line with Dutch oncology guidelines, a time to treatment of over six weeks was considered as delayed treatment [15].

### 2.3 Outcomes

The primary outcome was the difference in distribution of SES between the pandemic and pre-pandemic cohorts; representation of patients with low SES was used as a measure for access to surgical cancer care. Secondary outcomes were the likelihood of locally advanced tumour stage (Stage III based on pTNM) – used as a measure of delayed presentation – and time to treatment initiation – explored as a potential proxy for patients’ ability to navigate the healthcare system when facing scheduling challenges. Which may relate to health literacy rather than differences in provided care.

### 2.4 Missing data

Multiple imputation was used to handle missing data. A multiple imputation model was built including the variables associated with missingness or value of SES, and the other intended covariates and outcome variables of the planned logistic regression analysis. Predictive Mean Matching (PMM) was applied as imputation method for continuous variables such as SES, and logreg/polyreg for categorical variables. Imputation was repeated 15 times. Imputed data was compared to the complete cases to determine validity of the imputation model. For the analysis of the primary outcome - SES distribution - a complete-case analysis was performed on the original dataset. To evaluate the potential bias introduced by missing SES values, the results were compared with those obtained from the imputed dataset. For the analysis of the secondary outcomes, a dataset consisting of original data on the outcome variables was used, with imputation performed for missing values in the covariates - including SES. For all analysis on imputed data, model estimates and standard errors were calculated according to Rubin's rules [16].

### 2.5 Statistical analysis

Analyses combined patients across all included tumour types. Descriptive statistics were used to compare patient and tumour characteristics between the pre-pandemic and pandemic cohorts. Distribution of SES between the cohorts was assessed with a density plot, Wilcoxon rank sum test on numerical SES scores, and chi-squared test on SES groups. Multivariate logistic regression analyses were performed to study the association between SES and the likelihood of being operated for a stage III tumour, and the likelihood of treatment initiation later than 6 weeks from first visit. Covariates included sex, age, cardiac and pulmonary comorbidity, ASA score, and tumour type.

Data were analysed using RStudio version 4.2.3 (R Foundation for Statistical Computing, Vienna, Austria, 2023). The package gtsummary was used for creating tables, ggplot2 for creating graphs, and mice for multiple imputation.

## 3 Results

A total of 29,792 procedures were analysed: 14,208 in the pandemic cohort and 15,584 in the pre-pandemic cohort. The mean number of procedures per week decreased from 136 in the pre-pandemic cohort to 129 procedures in the pandemic cohort.

### 3.1 Patient characteristics

The mean age at the date of surgery was 66 years; 42% of patients were female and the majority (70%) was treated for a stage I or II malignancy (Table 1). These characteristics did not statistically differ between the pandemic and pre-pandemic cohorts. Surgical patients in the pandemic cohort less often had pulmonary comorbidity (19% vs. 21%, p = 0.02; but they were categorized with higher ASA scores (ASA 3: 40% vs. 36%, p < 0.001). Lung cancer was the most common malignancy in both the pre-pandemic and pandemic cohort.

**Table 1:** Patient characteristics.

Characteristic	Pre-pandemic cohort, N = 14,931^c^	Pandemic cohort, N = 13,545^c^	p-value^a^
Female sex	6296 (42%)	5718 (42%)	0.9
*Missing*	*<0.1%*	*0.1%*	
Age (years)	66 (20)	66 (11)	0.2 §
*Missing*	*0.2%*	*0.2%*	
Bmi >30	2243 (17%)	2060 (17%)	>0.9
*Missing*	*13%*	*13%*	
Pulmonary comorbidity	2899 (24%)	2621 (19%)	
*Missing*	*20%*	*0.7%*	
*Excl. 2018*	1663 (21%)	2621 (19%)	0.019
*Missing (Excl. 2018)*	*1.3%*	*0.7%*	
Cardiac comorbidity	1425 (12%)	1319 (9.8%)	
*Missing*	*21%*	*0.8%*	
*Excl. 2018*	805 (10%)	1319 (9.8%)	0.5
*Missing (Excl. 2018)*	*1.3%*	*0.8%*	
ASA score			<0.001
1	1046 (7.1%)	672 (5.1%)	
2	8120 (55%)	6988 (53%)	
3	5315 (36%)	5369 (40%)	
4	237 (1.6%)	241 (1.8%)	
*Missing*	*1.4%*	*2.0%*	
Tumour type			<0.001
Lung cancer	5742 (39%, 50/w)	4810 (36%, 44/w)	
Hepatic metastasis	2535 (17%, 22/w)	2341 (18%, 21/w)	
Oesophageal cancer	1768 (12%, 15/w)	1505 (11%, 14/w)	
Pancreatic cancer	1286 (8.8%, 11/w)	1434 (11%, 13/w)	
Gastric cancer	975 (6.7%, 8/w)	811 (6.1%, 7/w)	
Pulmonary metastasis	842 (5.8%, 7/w)	760 (5.7%, 7/w)	
Periampullary tumour	665 (4.5%, 6/w)	665 (5.0%, 6/w)	
Hepatocellular carcinoma	499 (3.4%, 4/w)	626 (4.7%, 6/w)	
Biliary tract tumour	326 (2.2%, 3/w)	330 (2.5%, 3/w)	
*Missing*	*2%*	*1.9%*	
Cancer stage^b^			0.4
I	3552 (40%)	3143 (40%)	
II	2686 (30%)	2377 (30%)	
III	2215 (25%)	1959 (25%)	
IV	456 (5.1%)	454 (5.7%)	
*Missing*	*18%*	*20%*	

Data are presented as mean (SD), and number (%).

a Pearson's Chi-squared test, except § Welch Two Sample *t*-test.

b Excluding procedures for hepatic or pulmonary metastasis.

c Unique patients.

### 3.2 SES distribution

**Fig. 1:**
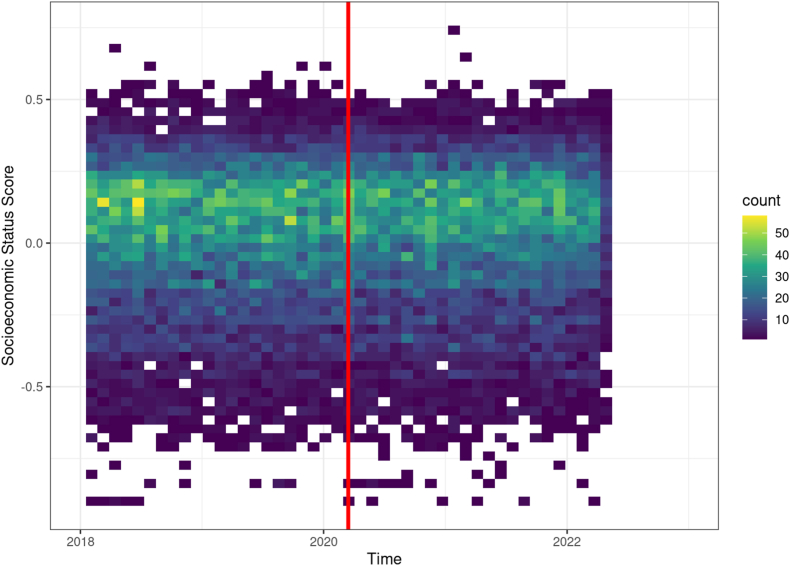
Distribution of SES scores over time.

The median SES score was comparable in the pre-pandemic and pandemic cohorts (0.057 and 0.059, p = 0.7). Similarly, the distribution of SES groups (Table 2) and the density heatmap (Fig. 1) did not show any differences between the pandemic and pre-pandemic cohorts (p = 0.6). Patients residing in high SES areas constituted 43% of the cohort, while those residing in low SES areas constituted only 17%. After multiple imputation, the median SES score did not change notably compared to the original data (Table 2).

**Table 2:** SES distribution.

	Pre-pandemic cohort	Pandemic cohort	p-value^a^
**Original data**			
Socioeconomic status score (range −1 to 1)	0.057 (−0.119, 0.184)	0.059 (−0.119, 0.187)	0.7
*Missing*	*16%*	*18%*	
Socioeconomic status category			0.8
Low	2166 (16%)	1964 (17%)	
Medium	5343 (41%)	4713 (40%)	
High	5651 (43%)	5035 (43%)	
*Missing*	*16%*	*18%*	
**Imputed data**			
Socioeconomic status score (range −1 to 1)	0.059 (−0.116, 0.185)	0.060 (−0.117, 0.187)	0.8
Socioeconomic status category			0.7
Low	16 %	17 %	
Medium	41 %	40 %	
High	43 %	43 %	

Data are presented as median (IQR) and number (%).

a Wilcoxon rank sum test; Pearson's Chi-squared test; Rubin's rules applied.

### 3.3 Locally advanced disease

**Fig. 2:**
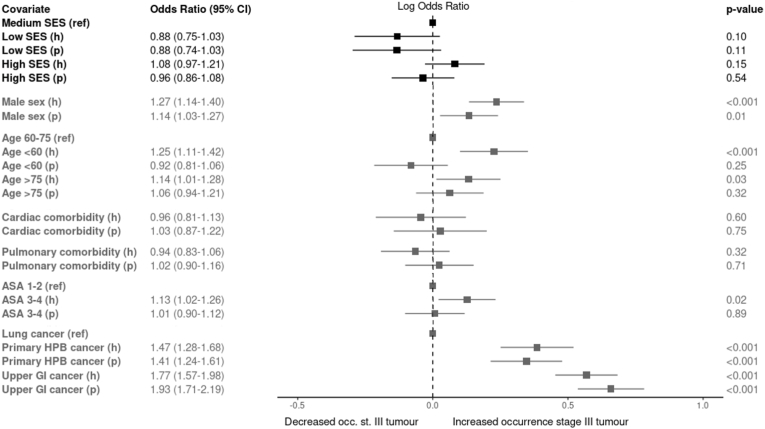
Forest plot: association of covariates to occurrence of surgeries for stage III tumours.

The distribution of tumour stages was similar in the pre-pandemic and pandemic cohorts (p = 0.4, Table 1); 25% of patients were operated for a stage III malignancy. A regression analysis showed that, in both the pre-pandemic and pandemic cohorts, patients with lower and higher socioeconomic status were equally likely to undergo surgery for a stage III tumour. After adjusting for patient characteristics and tumour type, lower SES was not associated with an increased occurrence of operations for locally advanced disease compared to the medium SES group, either in the pandemic cohort (aOR 0.88, 95% CI 0.74–1.03) or in the pre-pandemic cohort (aOR 0.88, 95% CI 0.75–1.03). Similarly, no significant difference was observed when comparing higher SES with medium SES. The results of the logistic regression are shown in Fig. 2. To take into account a possible delayed effect, a sensitivity analysis was performed excluding 2020. This showed similar results for the low SES group compared to the medium SES group (aOR 0.82, 95% CI 0.67 – 1.01).

### 3.4 Time to treatment

The median time between diagnosis and treatment was 27 days in both the pre-pandemic and pandemic cohorts (p = 0.1; unimputed data). A sub-analysis of time to treatment initiation in each of the three SES groups revealed no differences in time to treatment initiation between cohorts nor SES groups (Table 3). The occurrence of delayed treatments (>6 weeks between diagnosis and treatment initiation) was 23% in the pre-pandemic cohort and 22% in the pandemic cohort (p = 0.051). After correcting for patient characteristics and tumour type in logistic regression, patients with lower SES were statistically significantly more likely to face a treatment delay in the pre-pandemic cohort compared to the medium SES group (aOR 1.17, 95% CI 1.02 – 1.35). This disadvantage for patients with lower SES disappeared during the pandemic (aOR 0.98, 95% CI 0.85 – 1.13).

**Table 3:** Time to treatment according to SES (imputed data).

	Low SES		Medium SES		High SES	
	Pre-pandemic cohort,	Pandemic cohort,	Pre-pandemic cohort,	Pandemic cohort,	Pre-pandemic cohort,	Pandemic cohort,
**Time to treatment in days**	27 (16, 42)	27 (16, 39)	27 (16, 41)	27 (16, 40)	27 (16, 40)	27 (16, 40)
**p-value**^a^	0.23		0.12		0.39	

Delayed treatment	Pre-pandemic cohort				Pandemic cohort			
	rate	aOR	95% CI	p-value	rate	aOR	95% CI	p-value
**Low SES**	24%	1.17	1.02 – 1.35	**0.031**	20%	0.98	0.85 – 1.13	0.8
**Medium SES (ref)**	23%	-	-		22%	-	-	
**High SES**	22%	0.91	0.81 – 1.02	0.09	22%	0.97	0.86 – 1.09	0.7

a Pooled linear model.

### 3.5 Multiple imputation of missing data

The variable SES contained 17% missing data and displayed a Missing At Random (MAR) mechanism. Variables associated with missingness or value of SES were pulmonary comorbidity, ASA score and neo-adjuvant treatment (yes/no).

## 4 Discussion

### 4.1 Main finding of this study

This population-based cohort study of surgical oncology patients found no evidence of increased inequalities according to socioeconomic status in the context of healthcare scarcity during the COVID-19 pandemic. All three of our hypotheses were rejected. First, the distribution of socioeconomic status (SES) scores remained consistent, indicating that patients with lower SES continued to access surgical cancer care at rates comparable to the pre-pandemic period. Second, patients with lower SES were equally likely to undergo surgery for locally advanced tumours in both the pre-pandemic and pandemic cohorts, suggesting that delays in presentation were not disproportionately more frequent in this group. Third, the median time to treatment initiation did not differ significantly across SES groups, implying that patients with higher SES were not more successful in expediting their care.

### 4.2 What is already known on this topic and what this study adds

Although scientific interest in socioeconomic disparities in healthcare is increasing, the number of studies examining the impact of healthcare scarcity on these disparities remains limited. Several large-scale studies have used claims and reimbursement data to explore healthcare utilization across sociodemographic groups, though rarely in the field of oncology. Remarkably, and in contrast to our findings, most published research has reported widening inequalities during the COVID-19 pandemic [[17], [18], [19]]. To our knowledge, the only other study with findings similar to ours was conducted in Sweden, a country with an egalitarian healthcare system comparable to that of the Netherlands. Maresch et al. found that education level did not influence healthcare utilization during the pandemic for several essential services, including cancer diagnostics [20]. In contrast, Feras et al. reported disproportionately large reductions in healthcare utilization during the pandemic in socioeconomically deprived areas of Hungary [17]. And Gyeltshen et al. found worsened inequalities in cancer screening participation in Japan [18]. In the Netherlands, Frey et al. analysed reductions in non–COVID-19 healthcare and found that these were unequally distributed across sociodemographic groups, being more pronounced among individuals living below the poverty line, women, older people, and those with a migration background [19]. For oncology, however, they observed minimally higher reductions among individuals living below the poverty line. This may partly explain the discrepancy between their results and ours.

Our secondary outcomes were the likelihood of locally advanced tumour stage and time to treatment initiation. For both, we observed no increase in inequalities during the pandemic. A literature search revealed no other publications addressing disparities in disease stage in this context. The aforementioned study by the Netherlands Comprehensive Cancer Organisation reported more favourable disease stages at diagnosis for higher-income groups, but only for certain tumour types (cervical, breast, rectal, prostate, and melanoma), often in relation to cancer screening programs [7]. None of these tumour types were included in our analysis, which may explain the absence of socioeconomic disparities in both the pre-pandemic and pandemic cohorts. Other studies have investigated disparities in time to treatment. Two large U.S. studies reported that lower income and non-White race were associated with longer delays to surgery [21,22]. In contrast, another U.S. study found no racial or socioeconomic disparities in timely breast cancer treatment among Military Health Service beneficiaries [23]—a population more comparable to ours, with health insurance coverage and smaller economic differences than in the general U.S. population.

Given the limited number of published studies reporting no widening of inequalities, publication bias may be influencing the evidence base. Beyond potential publication bias, several plausible explanations may account for why our findings differ from prior studies. The potential for inequality is likely lower in countries such as the Netherlands, which have universal healthcare systems, compared with systems characterized by private insurance and private healthcare providers. Nonetheless, navigating the Dutch healthcare system is still easier from a position of privilege, and socioeconomic disparities in cancer care do exist, as demonstrated by the Netherlands Comprehensive Cancer Organisation. Our initial hypothesis was that healthcare scarcity during the COVID-19 pandemic would exacerbate barriers to access and disproportionately affect lower socioeconomic groups. It is possible, however, that these barriers were less severe than expected. Frey et al. did report an uneven toll of the pandemic on the general Dutch population [19], but oncology services may have been relatively protected, as cancer care was prioritized over other types of healthcare. When postponement of care is limited, the potential for unequal distribution of the burden is also reduced. Another explanation might lie in the scope of our dataset, which included only patients who underwent surgical treatment. It is known that patients with lower SES less often receive cancer treatment with curative intent such as surgery [7]. Increased disparities in cancer care may have occurred outside the scope of surgical oncology and it is possible that they were therefor not captured in our study. Taken together, our findings indicate that among patients with cancer who underwent surgery, there was no evidence of increased socioeconomic disparities during the pandemic. However, disparities in non-surgical treatment could not be assessed.

### 4.3 Strengths and limitations of this study

A major strength of this study is the use of data from national mandatory quality registries that include all patients who underwent surgery for lung, HPB, and upper gastrointestinal cancers up to the end of the pandemic. These datasets provide high detail and assured data quality [13]. Multiple imputation was applied to minimize bias from missing data. Socioeconomic status was derived from postal code and thus reflects neighbourhood-level rather than individual-level characteristics; however, given the large sample size, this measure can be considered a reasonable proxy. Furthermore; neighbourhood SES has an effect in addition to individual SES, Guadamuz et al. [24] found that patients residing in lower SES area's had worse survival across the entire cohort; this effect did not increase but did persist during the COVID-19 pandemic. Although our outcomes were selected as three indicators of access to surgical cancer care, they cannot capture all dimensions of health inequality. The datasets of the quality registries we used in the study included only surgically treated patients; therefor the tumour stage distribution cannot accurately represent the entire population of patients with cancer in the Netherlands in the pandemic group, nor in the pre-pandemic group. In addition, the increased use of neoadjuvant therapy during the pandemic may have affected the number of patients undergoing surgery for locally advanced tumours. Stage migration due to delayed presentation or diagnosis would not be expected within the first year of the pandemic. Therefore, we conducted a sensitivity analysis excluding data from 2020, which did not alter our conclusions.

### 4.4 Conclusions

Despite the unprecedented pressure on the healthcare system during the COVID-19 pandemic, this study found no evidence of increased socioeconomic inequalities in surgical cancer care in the Netherlands. These findings contrast with reports from other countries and suggest that the organization of a healthcare system determines the extent to which countries are vulnerable to rising inequalities in times of healthcare scarcity. It appears that healthcare systems such as that of the Netherlands — characterised by (near-) universal health coverage and the absence of parallel public–private systems or pronounced regional disparities in service availability — may be less susceptible to such inequalities.

Nonetheless, even within (near-)universal healthcare systems, scarcity can still give rise to disparities — also in oncology — whenever prioritization of cancer care becomes unfeasible. We therefore urge policymakers to safeguard equitable access to care and encourage healthcare professionals to remain attentive to the needs of marginalized populations. Furthermore, we call upon researchers to publish their findings even when no disparities are observed, as this will enhance our collective understanding of the factors that make healthcare systems resilient to threats against equity of access.

## Ethical statement

According to the Dutch Medical Research Involving Human Subjects Act (WMO - Wet medisch-wetenschappelijk onderzoek met mensen), research that uses only anonymized data where individuals cannot be identified directly or indirectly, and where no new procedures are performed on participants, falls outside the scope of the WMO. As this study involved retrospective analysis of existing anonymized registry data without any intervention or contact with patients, ethical approval was not required under Dutch law.

## Declaration of generative AI and AI-assisted technologies in the writing process

During the preparation of this work the author(s) used Microsoft Copilot for editing in order to improve readability. After using this tool/service, the author(s) reviewed and edited the content as needed and take(s) full responsibility for the content of the publication.

## Funding

This study was conducted within the project COVID Surg III, which is funded by 10.13039/501100001826ZonMw, an independent government body in the Netherlands (grant number 05160482110001).

## Declaration of competing interest

The authors declare the following financial interests/personal relationships which may be considered as potential competing interests: J. de Vos-Geelen reports a relationship with Servier Monde that includes: funding grants. S.S. Gisbertz reports a relationship with Medicaroid, J&J, Olympus that includes: consulting or advisory. Other authors declare that they have no known competing financial interests or personal relationships that could have appeared to influence the work reported in this paper.

## Data Availability

The data underlying this article were provided by the Dutch Institute of Clinical Auditing (quality registries Dutch Hepato Biliary Audit, Dutch Upper GI Cancer Audit and Dutch Pancreatic Cancer Audit) by permission. Data will be shared on request to the corresponding author only after permission of the respective quality registries.
